# Uncovering Bax inhibitor-1 dual role in the legume–rhizobia symbiosis in common bean roots

**DOI:** 10.1093/jxb/ery417

**Published:** 2018-11-21

**Authors:** Alejandrina Hernández-López, Mauricio Díaz, Jonathan Rodríguez-López, Gabriel Guillén, Federico Sánchez, Claudia Díaz-Camino

**Affiliations:** Departamento de Biología Molecular de Plantas, Instituto de Biotecnología, Universidad Nacional Autónoma de México, Avenida Universidad, Colonia Chamilpa, Cuernavaca, Morelos, Mexico

**Keywords:** Bax inhibitor-1, hypersensitive response, legume–rhizobia symbiosis, *Phaseolus vulgaris*, plant immune response, plant programmed cell death, plant autophagy, rhizobial endosymbiosis

## Abstract

Bax-inhibitor 1 (BI-1) is a cell death suppressor conserved in all eukaryotes that modulates cell death in response to abiotic stress and pathogen attack in plants. However, little is known about its role in the establishment of symbiotic interactions. Here, we demonstrate the functional relevance of an *Arabidopsis thaliana* BI-1 homolog (*PvBI-1a*) to symbiosis between the common bean (*Phaseolus vulgaris*) and *Rhizobium tropici*. We show that the changes in expression of *PvBI-1a* observed during early symbiosis resemble those of some defence response-related proteins. By using gain- and loss-of-function approaches, we demonstrate that the overexpression of *PvBI-1a* in the roots of common bean increases the number of rhizobial infection events (and therefore the final number of nodules per root), but induces the premature death of nodule cells, affecting their nitrogen fixation efficiency. Nodule morphological alterations are known to be associated with changes in the expression of genes tied to defence, autophagy, and vesicular trafficking. Results obtained in the present work suggest that BI-1 has a dual role in the regulation of programmed cell death during symbiosis, extending our understanding of its critical function in the modulation of host immunity while responding to beneficial microbes.

## Teaser

BaxI-1, a cell death suppressor that modulates cell death induction in response to abiotic stress and pathogen attack in plants, is also involved in shaping the legume–rhizobia symbiosis.

## Introduction

Common bean (*Phaseolus vulgaris* L.) seeds are a major source of carbohydrates, protein and essential micronutrients for populations in eastern Africa and Latin America (Namugwanya *et al.*, 2014). As a legume, common bean can enter into a mutualistic relationship with nitrogen-fixing rhizobacteria. This interaction results in the formation of a new organ in the root, the nodule, where rhizobia convert atmospheric nitrogen into ammonia to provide organic nitrogenous compounds to the plant.

Legume–rhizobia symbiosis is initiated under soil nitrogen-limiting conditions, when legumes attract host-specific rhizobial strains by the production of phenolic compounds and their exudation to the rhizosphere. These compounds prompt rhizobia to release nodulation factors (NFs) and attach to the surface of root hairs in active growth. After contact, the symbiotic signalling pathway is activated, inducing developmental responses such as division of pericycle and cortical cells to form the nodule primordium as well as major deformations of root hair extension, leading to bacterial invasion (Ferguson *et al.*, 2010; Oldroyd, 2013; Udvardi and Poole, 2013; Downie, 2014). The root rhizobial infection process occurs by the formation of a host-derived inwardly growing tubular compartment, known as the infection thread (IT), which guides the bacteria toward nodule primordium cells (Fournier *et al.*, 2015). Rhizobia are released from the ITs into the cytoplasm of developing nodule cells by an exocytosis-like pathway (Ivanov *et al.*, 2012), where they remain surrounded by a membrane called the symbiosome membrane. Within this membrane, rhizobia differentiate into bacteroids able to fix nitrogen. Bacterial differentiation is concomitant with host cell enlargement coupled to repeated endoreduplication cycles, which result in large polyploid cells housing thousands of bacteroids (Cermola *et al.*, 2000; Kondorosi *et al.*, 2000; Jones *et al.*, 2007).

A growing body of evidence suggests that a balanced regulation of the plant’s innate immunity is required throughout rhizobial infection, symbiotic accommodation into the nodule cell, and maintenance (Kouchi *et al.*, 2004; Lohar *et al.*, 2006; Brechenmacher *et al.*, 2008; Libault *et al.*, 2010; Berrabah *et al.*, 2015; Gourion *et al.*, 2015; Cao *et al.*, 2017; Zipfel and Oldroyd, 2017). Therefore, investigating the function of genes traditionally related to defence responses in legume–rhizobia symbiosis could improve our knowledge of legume biology.

Bax inhibitor I (BI-1) is an evolutionarily conserved transmembrane protein predominantly localized in the endoplasmic reticulum (ER) (Ishikawa *et al.*, 2011). BI-1 activity has been associated to the suppression of the hypersensitive response (HR), a well-characterized form of cell death that occurs during the plant immune response (Iwata and Koizumi, 2005; Watanabe and Lam, 2008, 2009). By adjusting the steady-state level of Ca^2+^ in the ER during stressful conditions (Kim *et al.*, 2008), BI-1 also promotes autophagy, a self-degrading cellular process with adaptive functions (Castillo *et al.*, 2011; Xu *et al.*, 2017).

Several plant transgenic approaches have shown that the loss of BI-1 function results in a severe programmed cell death (PCD) phenotype under abiotic stress (Watanabe and Lam, 2008, 2009; Duan *et al.*, 2010), whereas BI-1 overexpression attenuates plant PCD caused by the attack of pathogens (Matsumura *et al.*, 2003; Kawai-Yamada *et al.*, 2004; Babaeizad *et al.*, 2008; Watanabe and Lam, 2008, 2009; Isbat *et al.*, 2009; Ishikawa *et al.*, 2010). However, little is known about its role in symbiotic interactions. In the present study, we explore the biological function of a BI-1 homolog (*Pv*BI-1a) in the symbiosis between the common bean and *Rhizobium tropici*. *Pv*BI-1a is transiently induced in the plant root after contact with rhizobia. Overexpression of *PvBI-1a* in roots of composite common bean plants promotes rhizobial infection during early symbiosis, but induces the premature death of symbiotic nodule cells, thus diminishing the nitrogen-fixing ability of young nodules. At the molecular level, the overexpression of *PvBI-1a* affects the expression of defence, autophagy, and vesicular trafficking machinery genes; these cellular processes are known to be essential for the success of diverse plant–microbe interactions (Yuk *et al.*, 2012; Lamb *et al.*, 2013; Stolz *et al.*, 2014; Estrada-Navarrete *et al.*, 2016).

## Materials and methods

### Bacteria and plant material

*Escherichia coli* DH5α and *Agrobacterium rhizogenes* K599 (Bond and Gresshoff, 1993) were grown at 37 °C and 30 °C, respectively, in Luria–Bertani (LB) medium (Thermo Fisher Scientific, Waltham, MA, USA) supplemented with the appropriate antibiotics. *Rhizobium tropici* CIAT899 (Martínez-Romero *et al.*, 1991), *R. tropici* CIAT899–Discosoma red fluorescent protein (DsRed), and *R. tropici*–green fluorescent protein (GFP) were grown at 30 °C for 2 d in peptone-yeast extract (PY) medium, as previously described (Martínez-Romero *et al.*, 1991).

Surface-sterilized seeds of common bean (*Phaseolus vulgaris* cv. Negro Jamapa) were germinated on water-saturated paper towels in the dark at 28 °C for 2 d. The bean seedlings were then transferred to pots containing vermiculite and were inoculated with 1 ml of *R. tropici* diluted to an OD_600_ of 0.05 (equivalent to 7 × 10^7^) in 10 mM MgSO_4_ (Martínez-Romero *et al.*, 1991). The plants were grown in a glasshouse with a controlled environment (26–28 °C, 16 h photoperiod) and watered with Fahraeus nutrient solution (Fahraeus, 1957). Control plants (non-inoculated with rhizobia) were irrigated with Fahraeus nutrient solution supplemented with 8 mM KNO_3_ and grown in the same conditions). Alternatively, bean composite plants with transgenic roots were generated as described (Estrada-Navarrete *et al.*, 2006) and *R. tropici* inoculated as described. Wild-type or transgenic roots of common bean plants inoculated or non-inoculated with *R. tropici* were individually collected at specific time points, frozen in liquid nitrogen, and ground to a fine powder. Samples were stored at −80 °C until use.

### Plasmid construction and plant transformation

Two *BI-1* genes (*PvBI-1a* and *PvBI-1b*) with high sequence similarity to Arabidopsis *BI-1* (Sanchez *et al.*, 2000) were identified in the genome of common bean (Goodstein *et al.*, 2012). Using PCR, both genes were successfully amplified from a collection of full-length cDNAs generated from common bean. In addition, 1 kb of each of the *PvBI-1a* and *PvBI-1b* promoters was amplified from the common bean genomic DNA (see Supplementary Table S1 at *JXB* online). All DNA fragments were cloned into pENTR/D/TOPO cloning vectors (Thermo Fisher Scientific). The *PvBI-1a* overexpression construct was generated by combining the pENTR/D/TOPO::*PvBI-1a* entry vector with the Gateway-compatible plant destination vector pEarleyGate103. In the resulting construct (35S:*PvBI-1a*), *PvBI-1a* was fused in-frame to GFP (Earley *et al.*, 2006). To produce the *PvBI-1a* silencing construct, 155 nt from the *PvBI-1a* 3′-untranslated region (UTR) was PCR-amplified and cloned into pENTR/D/TOPO. This vector was then combined with the pTdT-RNAi plant destination vector (Valdés-López *et al.*, 2008), which includes the ‘Tomato’ fluorescent protein (tdTomato protein) as a reporter, to generate the construct *PvBI-1a*-RNAi. Finally, 1 kb of the *PvBI-1a* or *PvBI-1b* promoter region was cloned into the pENTR/D/TOPO cloning vector and transferred into pBGWFS7 (p*PvBI-1a*:pBGWFS7 or p*PvBI-1-1b*:pBGWFS7), to produce GFP and β-glucuronidase (GUS) reporter fusions (Karimi *et al.*, 2002). Empty destination vectors (with the exception of pTdT-RNAi, which includes a nucleotide-scrambled sequence, Sac), served as negative controls in all experiments. The resulting plasmids were introduced by electroporation into *A. rhizogenes* K599 and used for plant transformation.

### Acetylene reduction assays

The nitrogen fixation rate was determined using the acetylene reduction method (Vessey, 1994). At 18 d post-inoculation (dpi), the transgenic roots of the composite bean plants were transferred into glass bottles and sealed with rubber stoppers. Air was immediately withdrawn from the closed vial and replaced with acetylene to a final concentration of 10% of the gas phase. The samples were incubated for 60 min at room temperature, and ethylene production was determined using a Varian 3300 chromatograph (Agilent Technologies, Santa Clara, CA, USA), as previously described (Fuentes-Ramírez *et al.*, 1999). Speciﬁc activity was expressed as µmol^–1^ C_2_H_2_^–1^ g^–1^ nodule dry weight (DW) h^–1^. In these experiments, the nitrogen-fixation capacity of untransformed *A. rhizogenes* K599 root nodules was considered as 100%.

### Quantitative evaluation of root nodule bacteria

Common bean plants were grown and inoculated with *R. tropici* as described above. Nine root nodules were harvested from each 18-dpi plant, surface-sterilized by immersion in ethanol (95%, v/v) for 10 s, and then in sodium hypochlorite (10%, v/v) for 10 min, rinsed with sterile water five times and homogenized in 1.5 ml of PY medium containing 20 µg ml^–1^ nalidixic acid and 10 µg ml^–1^ tetracycline. Bacterial cultures were serially diluted and plated on PY medium plates with the appropriate antibiotics. Plates were incubated at 30 °C for 3 d and colony-forming units (CFU) were counted; 200 µl of the wash water used in the final rinse was plated in the same growth medium as negative control.

### RNA extraction and PCR assays

Total RNA was isolated from frozen plant material using the ZR Plant RNA Miniprep kit (Zymo Research, Irvine, CA, USA), following the manufacturer’s instructions. RNA quantity was measured spectrophotometrically and only high-quality RNA samples with a 260/280 ratio between 1.9 and 2.1 and a 260/230 ratio greater than 2.0 were used for the analysis. For reverse transcription, 3 µg total RNA was treated with DNaseI (DNaseI (RNase-free) Thermo Fisher Scientific), then 1.5 μg of the RNA was reverse-transcribed using the RevertAid H Minus First Strand cDNA Synthesis Kit (Thermo Fisher Scientific), according to the manufacturer’s instructions. The cDNA was quantified in 15 μl qPCR reactions using Maxima SYBR Green qPCR Master Mix (Thermo Fisher Scientific) containing 1 μl cDNA, performed on an iCycler iQ5 (Bio-Rad, Hercules, CA, USA). The cycling conditions were: 3 min at 95 °C; followed by 35 cycles of 20 s at 95 °C, 15 s at 55 °C, and data acquisition at 81 °C. A negative control reaction without a template was included for each primer combination (see Supplementary Table S1). The melting curve protocol began immediately after amplification and consisted of 1 min at 55 °C followed by 80 steps of 10 s each with a 0.5 °C increase in temperature at each step. The relative numbers for *C*_t_ of each gene were normalized to the *C*_t_ of the reference gene, *Elongation factor 1-α* (*PvEf1-α*) (Nicot *et al.*, 2005). *PvEf1-α* has been tested in our laboratory and is a stable gene reference for nodulation studies in common bean (data not shown). Data were analysed using iQ5 Optical System software (version 2.1, Bio-Rad).

### Statistical analyses

In all experiments, at least three biological samples were analysed and three technical repeats were performed for each biological sample. Obtained data were subjected to an unpaired Student’s *t*-test or ANOVA to identify significant changes in gene expression among the various plant conditions. The change in gene expression was the dependent variable. A *P* value of ≤0.05 or lower was considered as significant. ANOVA was followed by *post hoc* multiple comparison tests (Tukey, Welch or Sidak) depending on the data.

### Microscopy analysis

Transgenic roots and 18 dpi root nodules were hand-sectioned using double-edged razor blades. Sections were mounted on microscope slides in 0.1 M phosphate buffer, pH 7.4. All fluorescence images were taken using a confocal microscope (LSM510; Carl Zeiss, Oberkochen, Germany). *Z*-Projected confocal images were generated using Fluoview Viewer (Olympus Corporation, Shinjuku, Tokyo, Japan) and ZEN Black (Carl Zeiss).

For optical microscopy, control and 18 dpi *PvBI-1a* overexpression or RNAi nodules (six nodules per experiment) were fixed in a mixture of 2% formaldehyde and 0.4% glutaraldehyde in 0.1 M Na-cacodylate buffer, pH 7.2, at 4 °C for 16 h. The post-fixation was done in 1% osmium tetroxide for 2 h, and the samples were then dehydrated in a graded ethanol series. The samples were embedded in LR-White resin (London Resin Company, Reading, UK) and polymerized under ultraviolet light at –20 °C for 48 h. Semi-thin sectioning (0.5–1.0 mm) was performed using an ultramicrotome (Ultracut R; Leica Microsystems, Wetzlar, Germany), and the sections were stained with 0.1% toluidine blue. For transmission electron microscopy, samples were stained with uranyl acetate. Thin (60 nm) sections were prepared with an ultramicrotome (Ultracut R). The electron microscopy analyses were performed using an EM900 transmission electron microscope (Carl Zeiss) dual vision coupled camera system (Gatan, Inc., Pleasanton, CA, USA).

## Results

### *PvBI-1a* expression changes during the common bean–*R. tropici* symbiosis

We identified two homologs of Arabidopsis *BI-1*, *PvBI-1a* (Phvul.003G224400) and *PvBI-1b* (Phvul.002G001400), in the common bean genome (Goodstein *et al.*, 2012). Using PCR, we amplified these genes and their respective transcripts from common bean genomic DNA and full-length cDNA and sequenced the PCR products. The *PvBI-1a* sequence had an open reading frame of 738 nucleotides, preceded by a 251-bp 5′-UTR and followed by a 221-bp 3′-UTR; analysis of *PvBI-1b* revealed a 735-nt open reading frame, with a 5′-UTR of 152 bp and a 158-bp 3′-UTR (see Supplementary Fig. S1). The predicted *Pv*BI-1a and *Pv*BI-1b proteins exhibited 72% and 68% identity and 86% and 87% similarity, respectively, to Arabidopsis BI-1 (NP_199523.1). An analysis of membrane-spanning domains by Phyre2 (Kelley *et al.*, 2015) revealed seven transmembrane domains in both common bean BI-1 predicted proteins (Supplementary Fig. S1).

**Fig. 1.**
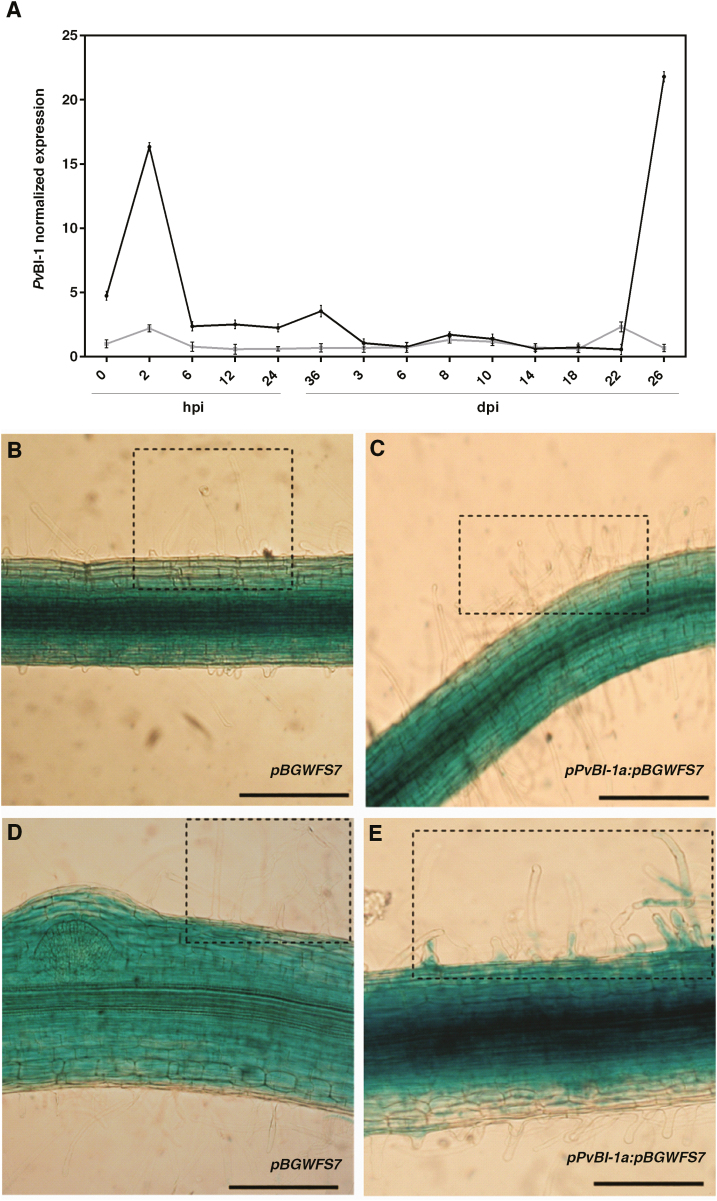
*PvBaxI-1a* expression is spatiotemporally modulated in common bean*–R. tropici* early symbiosis. (A) *PvBaxI-1a* (black line) and P*vBaxI-1b* (grey line) expression levels during nodulation determined using qPCR and normalized against *PvEf1-α* expression. The fold change in gene expression was obtained by comparing the expression ratio of each gene with its expression in uninoculated common bean roots. Plotted data are the mean values of transcript accumulation ±SD. The statistical significance was determined using two-way ANOVA (*P<0.005). (B–E) Representative images of the *PvBI-1a* promoter activity in GUS-stained common bean composite roots. (B, C) Uninoculated (B) or *R. tropici* inoculated (C) hairy-roots transformed with the pBGWFS7 vector. (D, E) Uninoculated (D) or *R. tropici* inoculated (E) p*Pv*BI1a:pBGWFS7-transformed hairy-roots. Photographs were taken 2 h after treatment. Root hairs are enclosed in squares. Scale bars, 200 μm.

We analysed the expression pattern of these two *PvBax-I* genes by qPCR throughout the common bean–*R. tropici* symbiosis. In these experiments, five plant roots were pooled and considered as one biological sample. The fold change in gene expression was obtained by comparing the expression ratio of each gene with its expression in uninoculated common bean roots. As shown in Fig. 1A, both genes were induced in response to *R. tropici*, although the amplitude of the *PvBI-1a* response was higher (16.3 ± 0.24-fold increase compared with uninoculated common bean roots) than that of *PvBI-1b* (2.2 ± 0.08-fold increase compared with uninoculated roots). At 6 hours post-rhizobia inoculation (hpi), the expression level of both genes declined for up to 22 days post-rhizobia inoculation (dpi) (Fig. 1A). Rhizobial colonization of the root and differentiation into nitrogen-fixing bacteroids are known to occur during this time (Cermola *et al.*, 2000). In wild-type common bean nodules, *PvBI-1a* reached its maximum expression level at 22–26 dpi (Fig. 1A), coinciding with the highest nitrogen-fixing activity of common bean nodules (Olivares *et al.*, 2011).

**Fig. 2.**
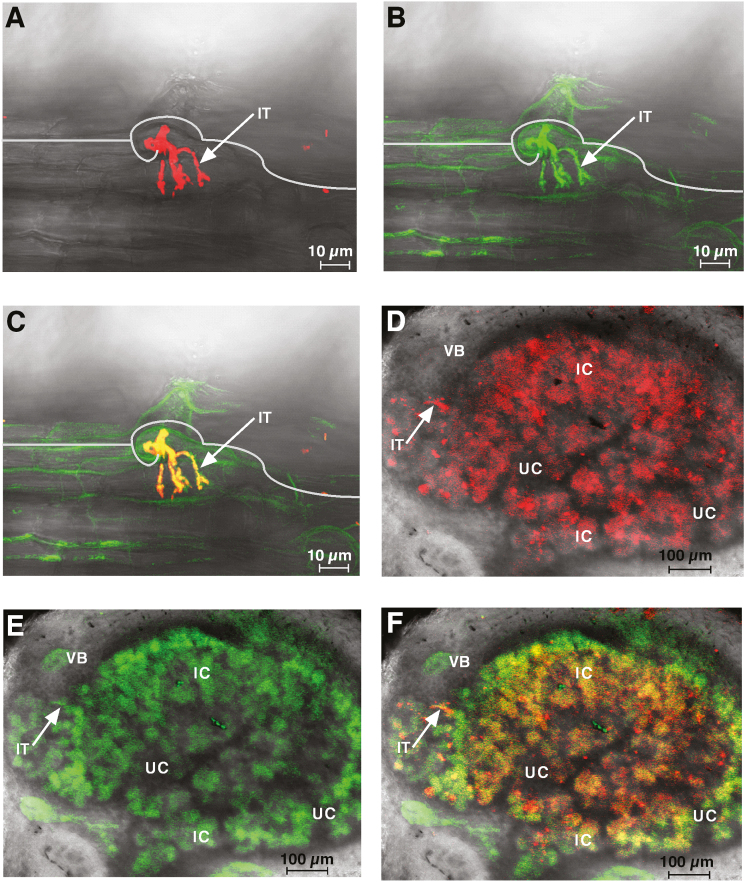
*PvBI-1a* promoter expression in symbiosis is restricted to ITs, symbiotic cells and vascular bundles of root nodules. (A) *R. tropici* CIAT899 DsRed migrating through infection threads at 3 dpi. (B) The activity of the *PvBI-1a* promoter is shown by the expression of GFP. (C) Merged image. (D) *R. tropici* CIAT899 DsRed in infected cells in an 18 dpi nodule. (E) Activity of the *PvBI-1a* promoter in this tissue. (F) Merged image. Representative images are shown. Transgenic roots or root nodules were analysed by confocal microscopy. Sixteen 1.89 μm optical sections were taken for each experimental condition. IC, infected cell; IT, infection thread; UC, uninfected cell; VB, vascular bundle.

To evaluate *PvBI-1a* and *PvBI-1b* promoter activity during nodulation, we cloned 1 kb of both promoters upstream of the translation initiation codon and fused them with GFP–GUS to yield the constructs p*PvBI-1a*:pBGWFS7 and p*PvBI-1b*:pBGWFS7. These constructs were used to generate transgenic roots in common bean plants (Estrada-Navarrete *et al.*, 2006). Transgenic roots harbouring the empty vector pBGWFS7 were used as a negative control. Five (*n*=5) independent GUS-stained roots of composite common bean plants uninoculated or 2 hpi with *R. tropici* were analysed. Representative images of these experiments are shown in Fig. 1B–E. The spatiotemporal expression of the *Pv*BaxI-1a promoter was detected along the root hair in response to *R. tropici*, as early as 2 hpi (Fig. 1E). The *Pv*BI-1a promoter activity was also monitored by GFP fluorescence in early symbiosis. At 3 dpi, GFP fluorescence was clearly detected in curled-hair roots and along ITs (Fig. 2A–C), while in nitrogen-fixing nodules at 18 dpi this promoter was active in the infected cells and in vascular bundles (Fig. 2D–F). However, no fluorescence signal could be detected during nodulation in *pPvBI-1b*:pBGWFS7 transgenic roots (data not shown). Given these results, we decided to focus our study on the functional characterization of *PvBI-1a*.

### *PvBI-1a* participates during *R. tropici* infection and its establishment in the root nodules of common bean

**Fig. 3.**
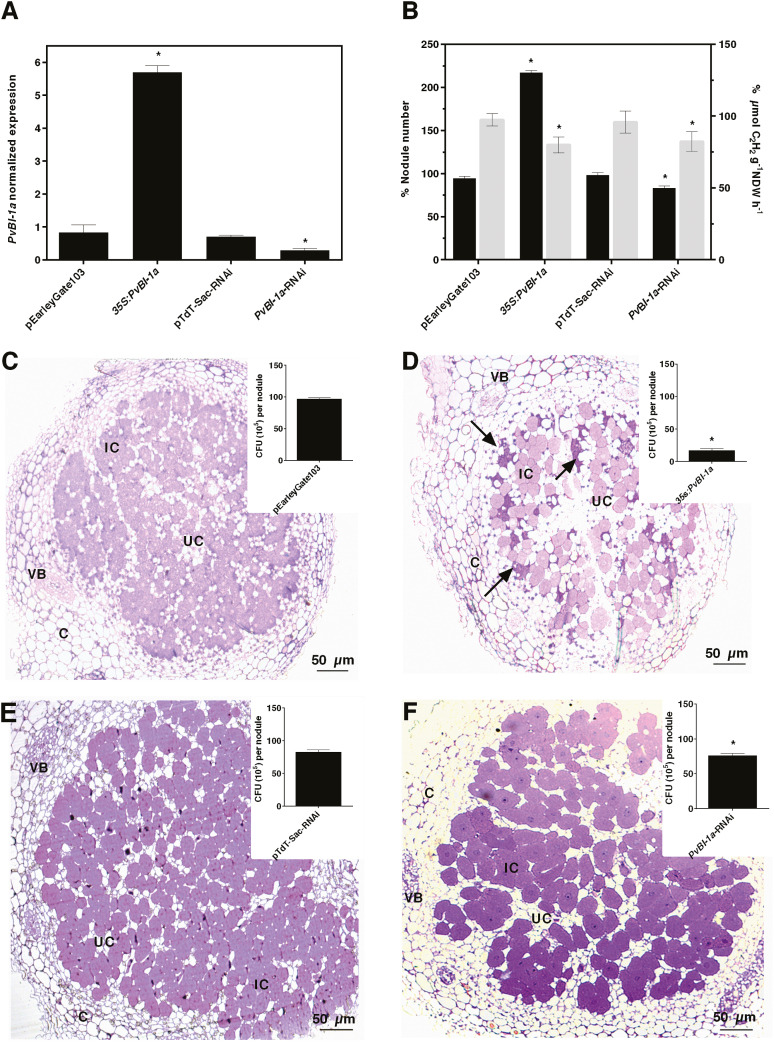
Changes in the expression level of *PvBI-1a* induce deleterious effects in nitrogen-fixing root nodules. (A) *PvBI-1a* transcript levels in 18 dpi *P. vulgaris* root nodules of pEarleyGate103 (negative control), 35S:*PvBI-1a*, pTdT-Sac-RNAi (negative control), and *PvBI-1a*-RNAi transformed roots determined by qPCR from three independent (*n*=3) biological replicates. Three technical repeats were used, and the data were normalized to the expression of *PvEf1-α*. Plotted data are the mean values of *PvBI-1a* transcript accumulation ±SD. (B) Total number of nodules formed per root (black columns) and their related nitrogenase activity (grey columns) in *P. vulgaris* transformed roots. Data are means ±SD from independent composite roots (*n=*20). Statistical significance in (A, B) was determined using an unpaired *t*-test followed by Welch’s correction (*P*<0.0001). (C–F) Optical microscopy of pEarleyGate103 (C), 35S:*PvBI-1a* (D), pTdT-Sac-RNAi (E), or *PvBI-1a*-RNAi (F) nodules. Representative images of six independent nodules are shown. C, cortex; IC, infected cells; UC, uninfected cells; VB, vascular bundle. Arrows in (D) indicate areas densely stained with toluidine blue. Insets in (C–F): *R. tropici* survival determined by colony-forming units (CFU) re-isolated from nodules. Values are means ±SD from nine (*n*=9) nodules of roots of composite plants, and statistical significance was determined with an unpaired *t*-test. (*P<0.0001). In all cases, 35S:*PvBI-1a* and *PvBI-1a*-RNAi data were normalized to associate values obtained from pEarleyGate103- and pTdT-Sac-RNAi-transformed roots, respectively.

To analyse the role of *PvBI-1a* in the common bean–*R. tropici* symbiosis, we altered *PvBI-1a* expression in roots by expressing it under the control of the 35S promoter (35S:*PvBI-1a*), or by decreasing its expression using RNA interference (*PvBI-1a*-RNAi). To this end, we produced common bean plants with transgenic roots using *A. rhizogenes*-mediated root transformation (Estrada-Navarrete *et al.*, 2006), either with the 35S:*PvBI-1a* or with *PvBI-1a*-RNAi constructs. Once generated, the transgenic roots of composite *P. vulgaris* plants were inoculated with equivalent *R. tropici* concentrations and the *PvBI-1a* transcript levels were determined in 18-dpi nodules using qPCR. In 35S:*PvBI-1a* nodules, *PvBI-1a* transcript levels or *Pv*BI-1a-GFP protein chimera levels were higher than in the negative control (pEarleyGate103-transformed nodules) (Fig. 3A and Supplementary Fig. S2, respectively), whereas expression levels in nodules harbouring *PvBI-1a*-RNAi were 2.4-fold lower than in the negative control (pTdT-Sac-RNAi, Fig. 3A).

The *PvBI-1a* loss-of-function nodules were similar in size and morphology (i.e. cortex and central tissue) to those of their corresponding negative control (empty vector pTdT-Sac-RNAi) (Fig. 3E–F), although the number of nodules formed on the *PvBI-1a*-RNAi roots (83.3 ± 4.2%) was slightly lower than the number of nodules found on the negative control (98.38 ± 2.5%; Fig. 3B). In contrast, *R. tropici* inoculation elicited 2.2-fold more nodules on the 35S:*PvBI-1a* roots (217.2 ± 4.4%) than on the empty vector pEarlyGate103 roots (94.5 ± 2.5%) (Fig. 3B). Compared with the negative control, 35S:*PvBI-1a* nodules were smaller in size, and had a diminished infection zone (Fig. 3C, D). As expected, their nitrogen fixation efficiency was considerably lower (~50% when considering total nodule number). However, this drop in the nitrogen fixation rate was compensated by the high number of nodules in the root (80 ± 5.5% compared with 97.4 ± 4.3 µmol C_2_H_2_ g^–1^ nodule DW h^–1^ in control nodules) so that it had no impact on plant growth (data not shown) (see Supplementary Fig. S3). We determined the number of viable bacteroids within all these nodules. The 35S:*PvBI-1a* and the *PvBI-1a*-RNAi root nodules had fewer viable bacteroids (16.8 ± 0.9% and 89.4 ± 1.0% CFU, respectively) compared with the negative controls (97 ± 0.6% in pEarleyGate103 and 97.6 ± 1.3% in pTdT-Sac-RNAi nodules) (inset graphs in Fig. 3C–F). While nitrogen fixation levels were lower in *Pv*BI-1a-overexpressing nodules compared with the negative control, the drastic reduction (~80%) in the number of free-living rhizobia recovered from these organs was unexpected. Presumably, the increased *Pv*BI-1a levels could be detrimentally affecting bacteroids inhabiting symbiotic cells. However, given that these nodules are morphologically distinct from *PvBI-1a*-RNAi and negative control nodules, we cannot rule out a technical bias arising from the sample preparation required for this analysis. Further experiments are necessary to investigate the cause of such a reduced recovery.

Since *PvBI-1a* and *PvBI-1b* were both expressed in *R. tropici*-inoculated common bean roots (Fig. 1A), we wondered whether the lack of a clear and unequivocal phenotype in *PvBI-1a*-RNAi nodules could be due to a functional redundancy with *PvBI-1b*. Thus, we determined the expression level of *PvBI-1b* in 10–26 dpi transgenic nodules and compared it with the negative control. As shown in Supplementary Fig. S4, the expression level of *PvBI-1b* was unaltered in 35S:*PvBI-1a* and *PvBI-1a*-RNAi nodules, indicating that *Pv*BI-1b was not likely to be compensating the function of *Pv*BI-1a in *PvBI-1a*-silenced nodules.

### *PvBI-1a* overexpression increases the number of rhizobial infection events in roots of composite common bean plants

**Fig. 4.**
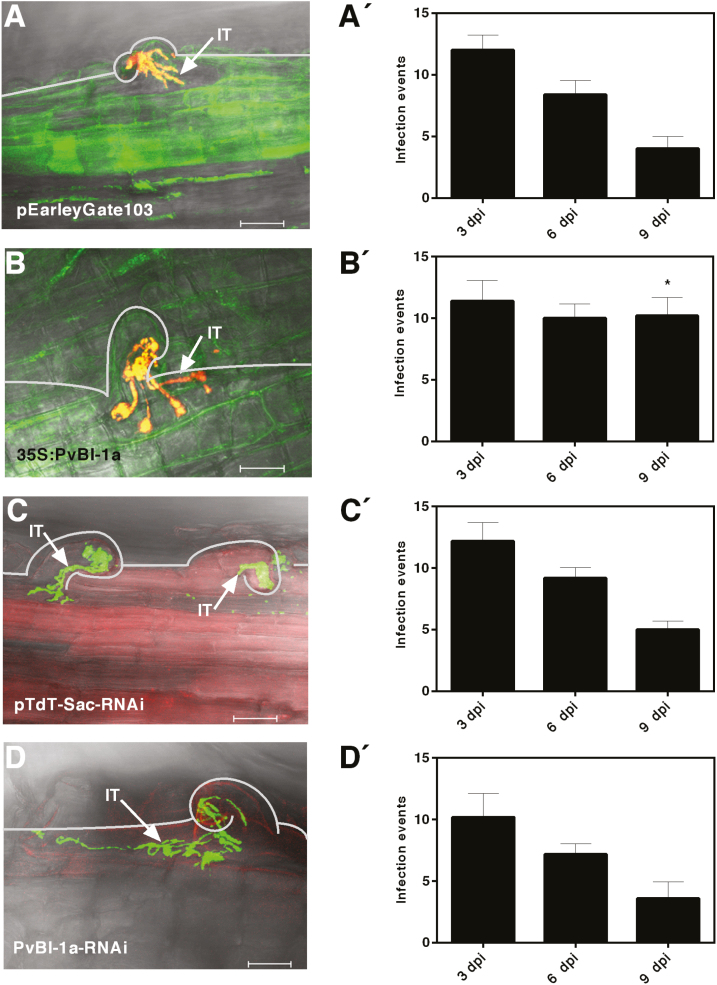
The overexpression of *PvBI-1a* in roots of composite common bean plants increases the frequency of *R. tropici* infection events through time. (A–D) Curled root hairs of transgenic roots inoculated with *R. tropici* migrating through ITs at 3 dpi. (A, B) pEarleyGate103- (A) or 35S:*PvBI-1a*- (B) transformed roots inoculated with *R. tropici*-DsRed. (C, D) pTdT-Sac-RNAi- (C) or *PvBI-1a*-RNAi- (D) transformed roots inoculated with *R. tropici*-GFP. Images were taken using a confocal microscope (LSM510; Carl Zeiss, Oberkochen, Germany). Sixteen 1.89 µm optical sections were taken for each experimental condition. *Z*-Projected confocal images were generated using Fluoview Viewer (Olympus Corporation, Shinjuku, Tokyo, Japan) and ZEN Black (Carl Zeiss). IT, infection thread. Bars, 20 μm. A′–D′ graphs depicting the number of curled root hairs with *R. tropici* migrating through ITs at 3, 6, or 9 dpi. Values are means ±SD from five (*n*=5) independent transgenic roots obtained from distinct plants, and statistical significance was determined with a two-way ANOVA followed by Tukey’s test (*P<0.05).

The number of nodules formed in a legume root host is locally controlled by the HR, which prevents IT formation, growth, and ramification (Vasse *et al.*, 1993). In consequence, the final number of nodules is programmed in early symbiosis. At this stage, the number of successful infection events can be determined by the number of curled root hairs with ramified ITs harbouring living rhizobia. Since the overexpression of *PvBI-1a* in transgenic roots of composite bean plants resulted in a dramatic rise in root nodule number (Fig. 3B), we determined the frequency of *R. tropici* successful infection events in *PvBI-1a* gain- or loss-of-function roots at different times (3, 6, and 9 dpi) (Fig. 4). As expected, the number of successful infection events in the negative control decreased over time after *R. tropici* inoculation (Fig. 4A′). The frequency of these events was sustained in *PvBI-1a*-overexpressing roots (Fig. 4B′). In contrast, silencing of *PvBI-1a* caused no significant differences over time compared with the control (Fig. 4C′, D′). Collectively, our results indicate that the overexpression of *PvBI-1a* in roots of common bean plants inoculated with *R. tropici* resulted in a higher number of infection events over time, thereby increasing the final number of nodules per root.

### Overexpression of *PvBI-1a* in common bean nodules induces premature cell death in symbiotic nodule cells

**Fig. 5.**
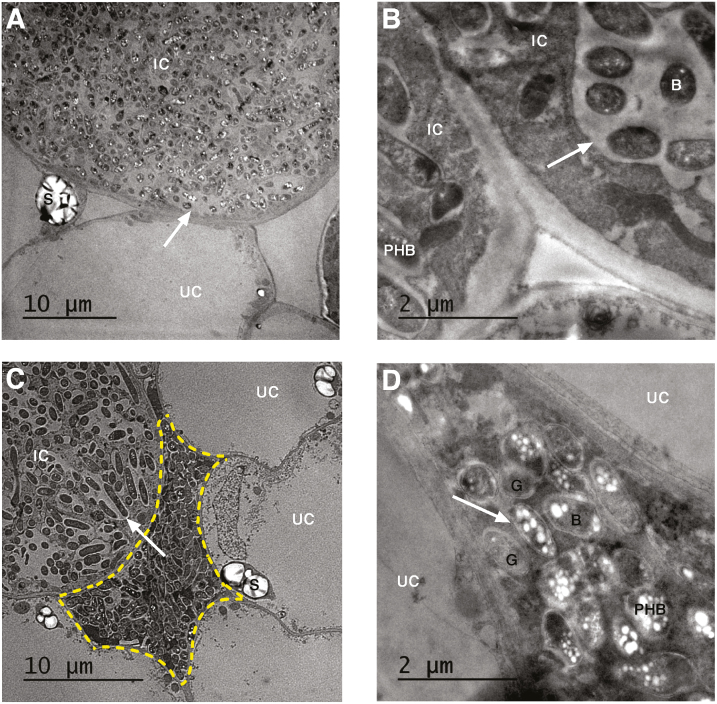
High expression levels of *PvBI-1a* induce premature senescence in young nodules of common bean. High magnification transmission electron micrographs of 18 dpi 35S:*PvBI-1a* root nodules inoculated with *R. tropici* at two magnifications. (A, B) Common bean root nodules transformed with pEarleyGate103. (C, D) 35S:*PvBI-1a P. vulgaris* root nodules. White arrows indicate the symbiosome frontier. Highlighted in yellow, a symbiotic nodule cell undergoing degradation. B, bacteroid; IC, infected cell; PHB, poly-3-hydroxybutyrate; S, starch grain; UC, uninfected cell.

During the microscopic analysis of 18 dpi *PvBI-1a*-overexpressing nodules, we detected areas between nodule cells that were densely stained with toluidine blue (Fig. 3D). These regions were further analysed using transmission electron microscopy (Fig. 5), which revealed the presence of old bacteroids (characterized by the overaccumulation of poly-β-hydroxybutyrate granules) as well as some degraded bacteroids (ghost, empty bacteroids) (Fig. 5C, D). No bacterial clusters were observed in control or *Pv*BI-1a-silenced nodules (see Supplementary Fig. S5). We assessed the level of cell death in *PvBI-1a*-overexpressing nodules by measuring the expression levels of a vacuolar processing enzyme (γVPE), and comparing them to those in *PvBI-1a*-RNAi and in negative control nodules. VPEs, and particularly γVPE, are caspase-like proteins that have been implicated in the execution of the ER-dependent PCD (Hatsugai *et al.*, 2004). Supplementary Fig. S6 shows γVPE induction in 18 dpi *PvBI-1a*-overexpressing nodules compared with the control, but not in *PvBI-1a*-RNAi nodules. This fact, in conjunction to the previously described morphological observations, suggests that at least some of the symbiotic nodule cells of 18 dpi *PvBI-1a*-overexpressing nodules are in PCD.

### *PvBI-1a* overexpression induces the premature death of symbiotic nodule cells

**Fig. 6.**
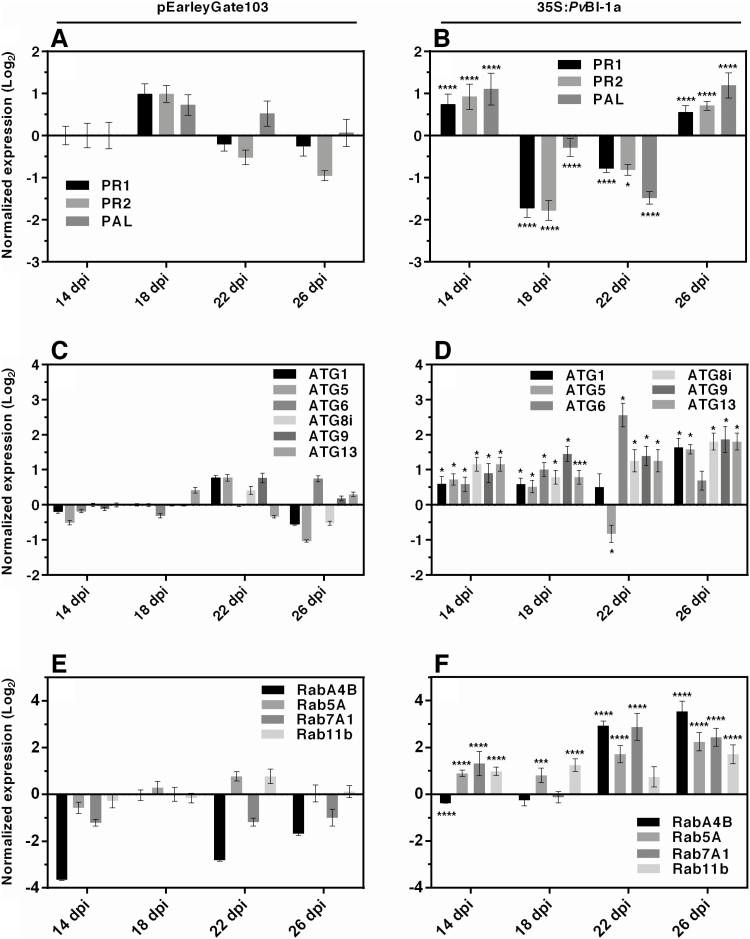
*PvBI-1a* overexpression induces the expression of defence, autophagy, and membrane trafficking genes during *P. vulgaris–R. tropici* symbiosis. mRNA levels of defence (A, B), autophagy (C, D), and membrane-trafficking (E, F) genes in 35S:*PvBI-1a* nodules (B, D, F) and in control nodules (A, C, E) of *P. vulgaris* composite plants 14–26 dpi with *R. tropici*, quantified using qPCR. Data from three independent (*n*=3) biological replicates (each with three technical replicates) were normalized to the expression of *PvEf1-α*. Plotted data are the mean log_2_ values of transcript accumulation ±SD. The statistical significance was determined using two-way ANOVA followed by Sidak’s multiple comparison test (*P<0.05; ***P<0.01; ****P<0.001).

Defence, autophagy, and membrane trafficking are fundamental cellular processes in plant–microbe interactions, including pathogenesis and symbiosis (Yuk *et al.*, 2012; Lamb *et al.*, 2013; Stolz *et al.*, 2014; Estrada-Navarrete *et al.*, 2016). We analysed the expression profile of a set of genes related to these interconnected cellular processes in control and *PvBI-1a* gain-of-function nodules (Fig. 6). Typically, the common bean nodule primordia may be unequivocally distinguished from lateral root primordia after 10 dpi in wild-type nodules. Given that the development of nodule primordia in common bean hairy roots inoculated with *A. rhizogenes* was slightly delayed, we decided to use 14–26 dpi nodules in these experiments. Nitrogen fixation levels from 22–26 dpi transgenic control nodules were similar (data not shown), indicating that rhizobia were fully differentiated to bacteroids after 22 dpi.

Compared with negative control nodules (pEarlyGate103 transformed root nodules), the onset of changes in the expression of defence-related genes occurred 4 d earlier in *PvBI-1a*-overexpressing nodules (Fig. 6A, B). In addition, the induction of core autophagy genes, including *ATG6*, which mediates PCD during the plant immune response in Arabidopsis (Liu *et al.*, 2005; Fig. 6D compared with control in Fig. 6C), and of genes tied to membrane trafficking (Fig. 6F compared with control in Fig. 6E) both support the pro-death role of BI-1 in 35S:*PvBI-1a* young root nodules. Compared with control (Supplementary Fig. S7 A, C, D), the silencing of *Pv*BI-1a in 18 dpi transgenic root nodules induces defence and most membrane trafficking genes (Supplementary Fig. S7B, F), but does not change the expression of autophagy-tied genes (Supplementary Fig. S6C–D).

## Discussion

While being mutually beneficial, rhizobial nodulation is energetically costly to the legume host, and it is known to be tightly controlled. Nodulation in legumes is regulated by local and systemic mechanisms. Locally, elicitation of the HR (a type of plant PCD) limits the number of infection events allowed, and therefore, contributes to determining the final number of active nitrogen-fixing nodules formed in the root of the legume host (Vasse *et al.*, 1993; Stacey *et al.*, 2006). Remarkably, the HR is also one of the earliest responses to pathogen attack in plants. The onset of the HR is associated with the synthesis of salycilic acid (SA), which induces extracellular calcium influx, production of reactive oxygen species (ROS), and *PR* gene expression (Grant *et al.*, 2000; Lam *et al.*, 2001; Hatsugai *et al.*, 2004). In parallel, SA also activates the expression of BI-1 (Lu *et al.*, 2018), whose expression is induced in biotic and abiotic types of cell death in plants (Kawai-Yamada *et al.*, 2001; Watanabe and Lam, 2008, 2009; Isbat *et al.*, 2009; Ishikawa *et al.*, 2010). Plant BI-1s have been implicated in ER stress-induced PCD and calcium homeostasis, and in promoting autophagy during pathogen attack (Kim *et al.*, 2008; Watanabe and Lam, 2008; Kawai-Yamada *et al.*, 2009; Duan *et al.*, 2010; Xu *et al.*, 2017).

Considering the relevant role of the HR in the context of legume nodulation, in this work we characterized the function of *Pv*BI-1a, a common bean homolog of Arabidopsis BI-1, throughout the process of symbiosis with *R. tropici*. As it occurs with ROS and some *PR* genes in early legume nodulation (Kouchi *et al.*, 2004; Lohar *et al.*, 2006, 2007; Brechenmacher *et al.*, 2008; Libault *et al.*, 2010), *PvBI-1a* expression transiently increased in common bean after rhizobial inoculation (Fig. 1A). A temporary increase of BI-1 expression after rhizobial inoculation has also been observed in soybean (Brechenmacher *et al.*, 2008), another tropical legume closely related to the common bean. The spatiotemporal induction of the *PvBI-1a* promoter in early symbiosis shows that this gene is being expressed in the root hair after rhizobial exposure, and during IT elongation (Figs 1B–E , 2A–C), precisely where the HR induces the abortion of a number of infection attempts (Vasse *et al.*, 1993). Given its role as a PCD suppressor in plants, these results lead us to hypothesize that during the early steps of the common bean–*R. tropici* interaction, *Pv*BI-1a could help modulate the HR, thus providing an additional step in defining the number of nodules formed in the root host. To test this hypothesis, we produced transgenic roots of composite common bean plants with either increased or decreased expression of *Pv*BI-1a (Fig. 3A; Supplementary Fig. S2). Both *PvBI-1a* variants were similar to control transgenic roots in aspects of hair root curling and IT development (Fig. 4A–D). However, the overexpression of *Pv*BI-1a increased the frequency of *R. tropici* infection events over time (Fig. 4A′–D′), and consequently, the final number of nodules formed in the root (Fig. 3B). These results support the notion that *Pv*BI-1a promotes rhizobial infection by attenuating the HR, and is consistent with previous reports where the susceptibility of diverse barley cultivars to *Blumeria graminis* f.sp. *hordei* (a biotrophic fungus) was found to correlate with higher BI-1 expression levels (Hückelhoven *et al.*, 2003; Babaeizad *et al.*, 2008), whereas in barley roots colonized by the mutualistic fungal endophyte *Piriformospora indica*, the expression of BI-1 is attenuated (Deshmukh *et al*., 2006).

Even though the final number of nodules formed in *PvBI-1a*-overexpressing roots was twice as large as the number on control roots (Fig. 3B), 35S:*PvBI-1a* nodules were smaller in size and poorly infected (Fig. 3C–D). Given that resident bacteroids inhabiting these organs were able to fix nitrogen (Fig. 3B), bacterial release from ITs to symbiotic nodule cells was likely not affected. A detailed analysis of the infection zone of *PvBI-1a*-overexpressing nodules revealed the presence of old or degraded bacteroids between uninfected or infected nodule cells (Fig. 5C–D). A similar phenotype has been observed in nodules of *Medicago truncatula activated defence 1* mutant plants (Wang *et al.*, 2016), where bacteroids and their symbiotic plant cells become necrotic as a consequence of an active plant defence. Such bacterial clusters were not observed in control or *PvBI-1a*-silenced nodules (see Supplementary Fig. S5). We confirmed the premature death of symbiotic cells in 35S:*PvBI-1a* nodules by assessing the expression level of γVPE in control or *PvBI-1a*-transgenic nodules. Because γVPE transcription increases during soybean nodule senescence (Roux *et al.*, 2014; Van Wyk *et al.*, 2014), γVPE induction in 35S:*PvBI-1a* nodules (Supplementary Fig. S6) suggests that these organs may be undergoing PCD. This is further supported by the premature gene activation observed during nodulation in 35S:*PvBI-1a* nodules not only of defence-related genes (Fig. 6A–B), but also of autophagy (Fig. 6C–D) and membrane trafficking genes (Fig. 6E–F). Induced expression of defence genes such as *PR1*, *PR2*, and phenylalanine ammonia lyase (PAL) have been also observed in plant senescence (Egli *et al.*, 1989; Quirino *et al.*, 1999; Morris *et al.*, 2000; Gourion, *et al.*, 2015) and it has been demonstrated that plant autophagy determines the resistance level against invading pathogens by controlling the amount of proteins involved in cell death through membrane trafficking events (Inada and Ueda, 2014; Leborgne-Castel and Bouhidel, 2014; Gourion, *et al.*, 2015; Ishikawa *et al.*, 2015). Additionally, Xu *et al.* (2017) recently demonstrated that BI-1 promotes autophagy during pathogenesis, establishing a direct connection between autophagy and plant resistance. Thus, the gene expression pattern we observed in 35S:*PvBI-1a* nodules also supports the notion that constitutive expression of *PvBI-1a* throughout the common bean–*R. tropici* symbiosis, induces premature PCD in symbiotic nodule cells. On the other hand, *Pv*BI-1a is highly induced in wild-type common bean nodules during active nitrogen fixation (Fig. 1A, 22–26 dpi nodules), although in this physiological context, where ROS are being intensively produced (Chang *et al.*, 2009), *Pv*BI-1a could potentially be playing a separate role related to antioxidant pathways. Such a function has been previously reported in mammals (Lee *et al.*, 2007; Kim *et al.*, 2009).

As we showed, the silencing of *PvBI-1a* gave no distinctive phenotype in early or late symbiosis). Since functional redundancy between BI-1 genes in common bean is unlikely (see Supplementary Fig. S4), these data suggest that other molecular signals involved in the positive control of nodule number, not yet identified, could compensate the lack of *Pv*BaxI-1a at this step of symbiosis. However, in later stages, a strict control of *Pv*BI-1a expression must take place to avoid the premature death of young nodules.

Overall, our results suggest that *Pv*BI-1a has a dual role during the legume–rhizobia symbiosis. While in early symbiosis BI-1 promotes rhizobial infection by repressing HR, in the later nodulation stages its overexpression leads to cell death, as occurs in other plant–microbe interactions (Xu *et al.*, 2017). By demonstrating the functional role of a BI-1 during nodulation in common bean, we have extended our understanding of the function of this protein in beneficial plant–microbe interactions.

## Supplementary data

Supplementary data are available at *JXB* online.

Fig. S1. *PvBI-1a* and *PvBI-1b* genes and sequence-deduced proteins.

Fig. S2. Overexpression of *Pv*BI-1a in root nodules of composite common bean plants.

Fig. S3. Control (pEarlyGate 103) and *PvBI-1a*-overexpressing composite bean plants inoculated with *R. tropici* CIAT899 (18 d later).

Fig. S4. Overexpression or silencing of *PvBI-1a* does not modify the expression level of *PvBI-1b* in common bean nodules.

Fig. S5. High magnification transmission electron micrographs of 18 dpi control and *Pv*BI-1a-RNAi root nodules show no disctintive phenotype.

Fig. S6. γVPE expression in 18 dpi *PvBI-1a* gain- or loss-of-function nodules.

Fig. S7. Silencing of *Pv*BI-1a in symbiotic nodules of common bean affects host defence and vesicular trafficking, but not autophagy.

Table S1. Oligonucleotides used in this study.

Supplementary_Material

## Abbreviations

BI-1: Bax inhibitor-1
dpi: days post-inoculation
ER: endoplasmic reticulum
hpi: hours post-inoculation
IT: infection thread
NF: rhizobial nod factor
PCD: programmed cell death.
