# Supplementary material for: Uncovering Bax inhibitor-1 dual role in the legume–rhizobia symbiosis in common bean roots
Source: J Exp Bot. 2018 Nov 21;70(3):1049–61. doi: 10.1093/jxb/ery417 (PMC6363093; doi:10.1093/jxb/ery417)
Supplement: Supplementary_Material [file ery417_suppl_supplementary_material.pdf]

Supplementary information

Gene name	Fw primer (5´to 3´)	Rv primer (5´to 3´)
Bax Inhibitor-1a (PvBI-1a)	ATGGAGGCTTTCTTCAATTCT<br>CAATCG	ATCTCTCCTTTTCTTCTTCC
Bax Inhibitor-1a (qPvBI-1a)	GGGAGTATCTCTACCTTGGTG<br>G	CCAGGTCACCAAAGTGAGC<br>C
Bax Inhibitor-1a (PvBI-1b)	ATGGATGCTTTCAATTCC	GACTGGTAATAAACTAATC<br>TCTCC
Bax Inhibitor-1b (qPvBI-1b)	GTGGAAGTTTCTGCGAAATTA<br>GG	ATCCATTGTTTTGGATCTCA<br>AACAC
Bax Inhibitor-1a-RNAi (PvBI-1a)	CACCATTCATCCGAGAGAAAT<br>GAGAGGAAG	CATGGCTAACAAACTACAC<br>ATGGGC
PvPR1	CTTCTGGCCAATCCCTGATTA<br>C	CACTCCTGAAAATGGAAGC<br>CAAC
PvPR2	AGTGGCTCCTGCTACCCTTTA<br>C	CTTTGTCTCCCCATCCTCAA<br>CG
PvPAL	GAGTACACATTTGCCTACATT<br>GAC G	GTTTTTCTCGTTCTCTCCAT<br>TGGC
PvATG1	GCAATACATTCACAAGTTGCA<br>GTCTCG	CCCCTTTACAAGGCCATGA<br>TAGTTG
PvATG5	GTTCGCATTCAAGGGATAAA<br>CCAG	TTATCCCTATTGCACGGCA<br>TTGGC
PvATG6	CTGCCGCAATCCTCTCTGCAT<br>C	CACACTGCTGGCACCATGA<br>ACG
PvATG8i	TGGGAGGAAGTTCAAGTTTCA<br>GGG	TCTTCTCCAACTCAGGCAG<br>ATCG
PvATG9	GCGACTTCCACATCCGTTCCC	CAATGACTGCTGGTAGTAT<br>GGGTC
PvATG13	CGCAACAGAATCAAAGATAA<br>GACCCTGC	GCCAAGAGCCTTCATATAA<br>TACTCG
PvRab4b	GTGAGAAAGAGAGTGGAAAT<br>GGCAG	CCTGGCTAGAATCTGCGAC<br>TTCC
PvRab5A	TGCCGAAGGAACTAGGGCTG<br>C	CCACATAACAGTGACATCA<br>AAGTGTGC
PvRab7A1	CAACAACCTTTAAGAGGAATC<br>CAGAAC	GAAATATCCATGTGGGAGA<br>CTCAAAAG
PvRab11b	CCATTCTGCTGCAAAAGGCTT	AGATTGGAGATGACCCTGC

	GTG	AGC
PvγVPE	GCA GAG AACTCC AGG GAG TTG C	GAG ACA CAT TGT GGA TCC CTG TC
Elongation factor-1a	GGTCATTGGTCATGTCGACTC TGG	GCACCCAGGCATACTTGAA TGACC
Bax Inhibitor-1a promoter (pPvBI-1a)	CCTTGGGAGAAGAATCTTGTA ATGG	TGATAAACAAGGAATCGTA TCGTTTTCG
Bax Inhibitor-1b promoter (pPvBI-1b)	GTA GAC CTA GTC TAA TGT GGA CC	TGTTTTGGATCTCAAACAC AAATTGG

Table S1.

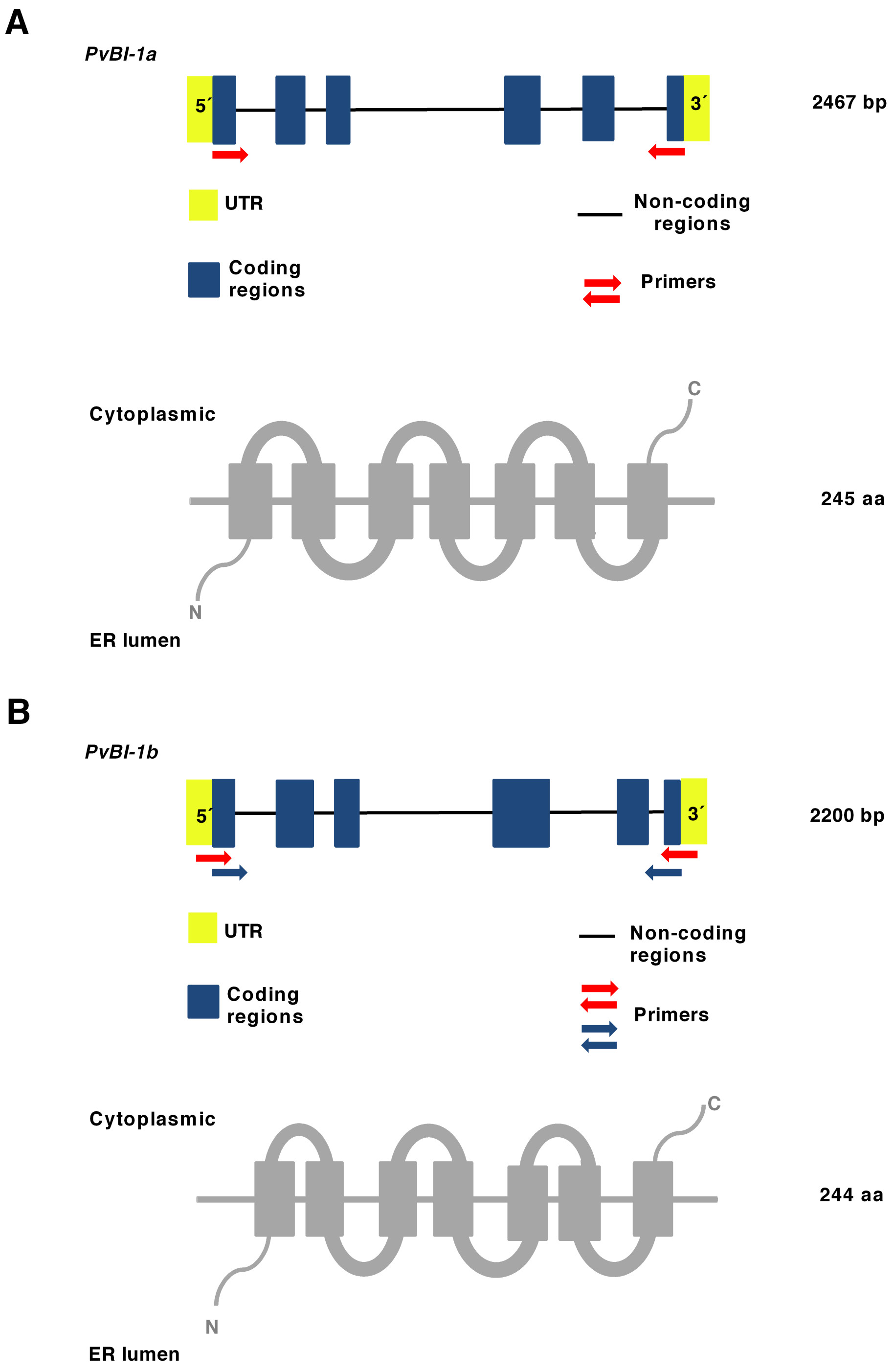
A
PvBI-1a
5´
3´
2467 bp
UTR
Non-coding regions
Coding regions
Primers
Cytoplasmic
C
245 aa
N
ER lumen
B
PvBI-1b
5´
3´
2200 bp
UTR
Non-coding regions
Coding regions
Primers
Cytoplasmic
C
244 aa
N
ER lumen

Fig. S1

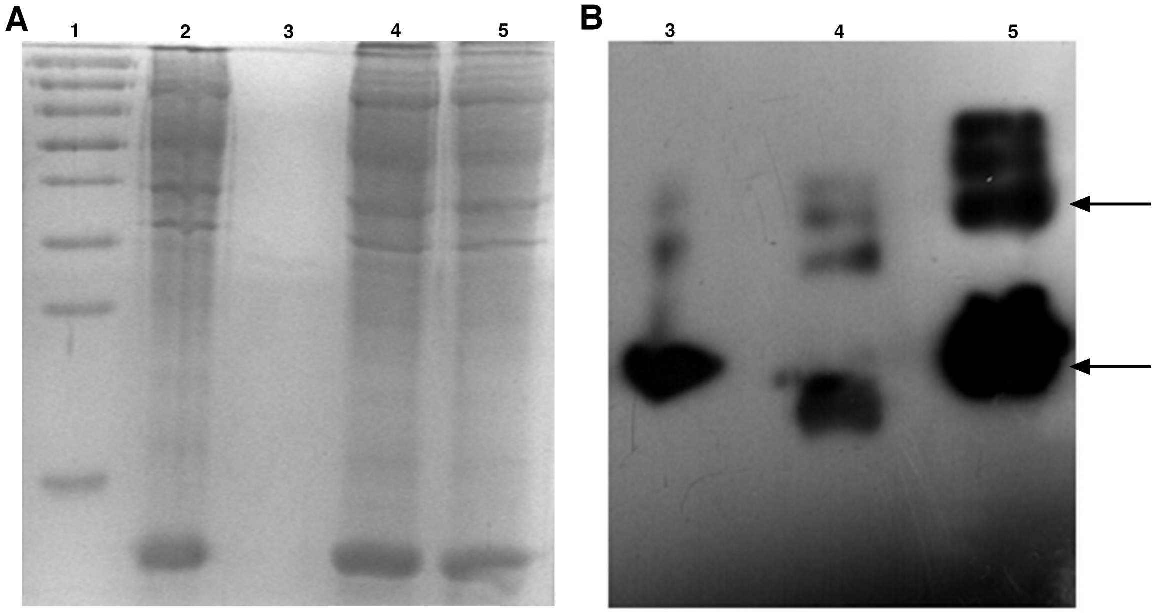

Fig. S2

pEarleyGate103 **35S:*Pv*BI-1a**

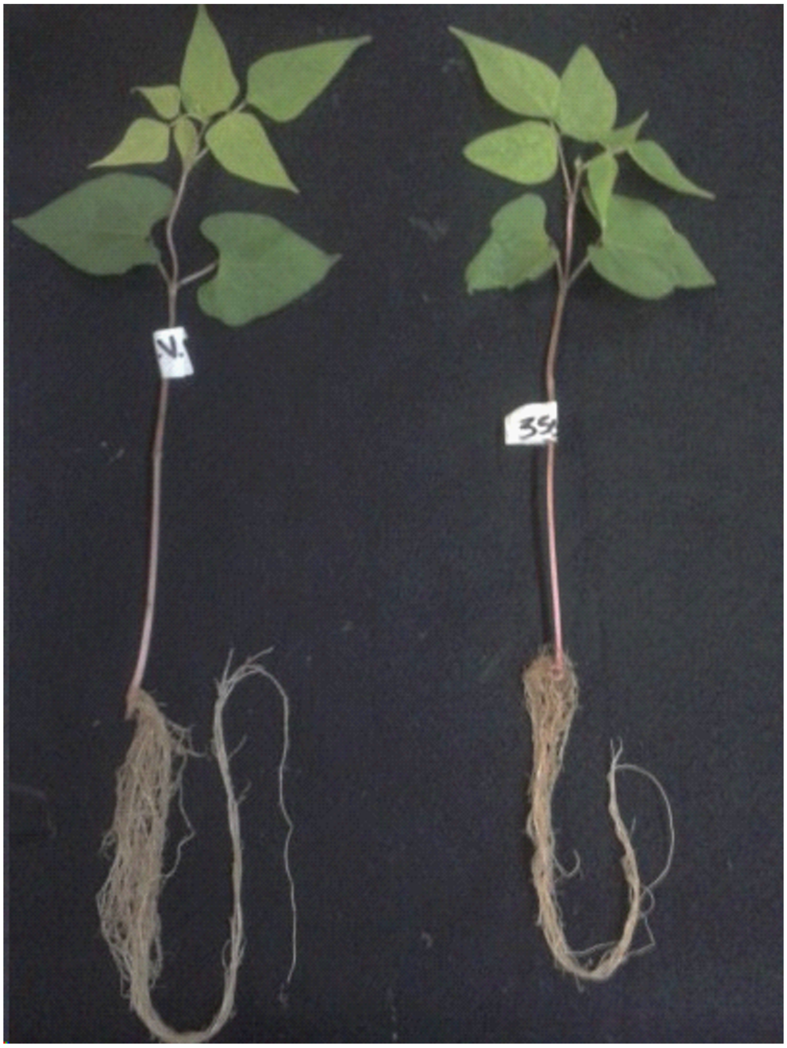

Fig. S3

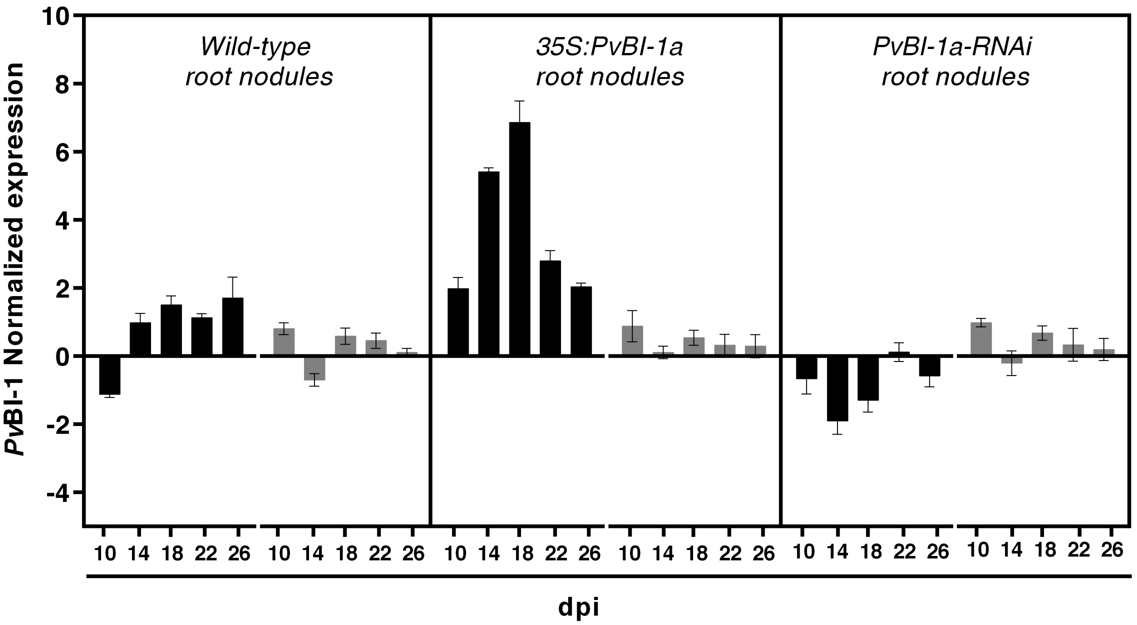

10
8
6
4
2
0
-2
-4
*Pv*BI-1 Normalized expression
Wild-type
root nodules
35S:PvBI-1a
root nodules
PvBI-1a-RNAi
root nodules
10 14 18 22 26 10 14 18 22 26 10 14 18 22 26 10 14 18 22 26 10 14 18 22 26 10 14 18 22 26
dpi

Fig. S4

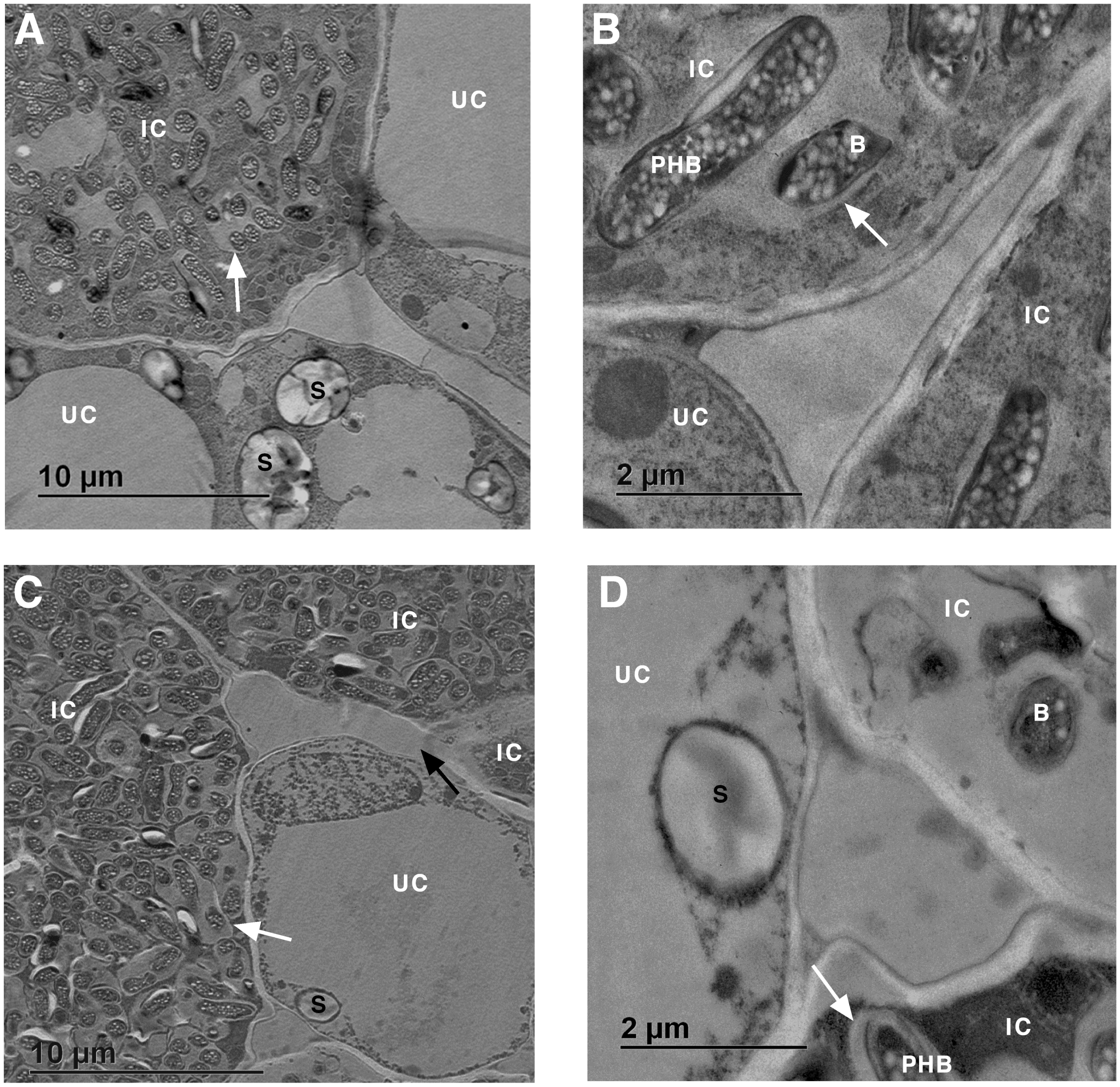
A
IC
UC
UC
S
S
10 µm
B
IC
PHB
B
IC
UC
2 µm
C
IC
IC
IC
UC
S
10 µm
D
IC
UC
B
S
2 µm
IC
PHB

Fig. S5

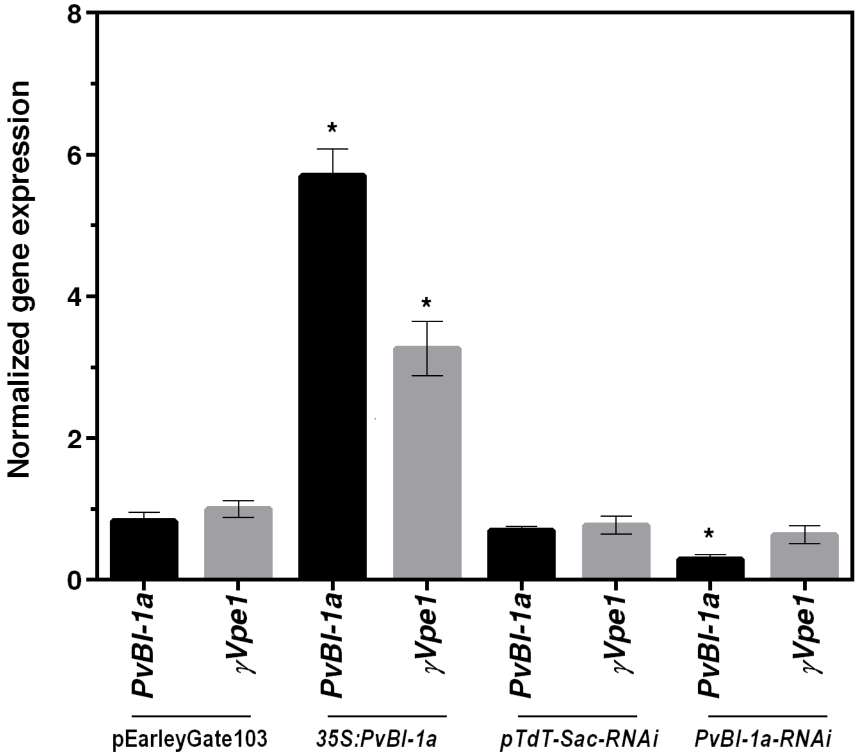
Normalized gene expression
8
6
4
2
0
*
*
*
PvBI-1a
γVpe1
PvBI-1a
γVpe1
PvBI-1a
γVpe1
PvBI-1a
γVpe1
pEarleyGate103
35S:PvBI-1a
pTdT-Sac-RNAi
PvBI-1a-RNAi

Fig. S6

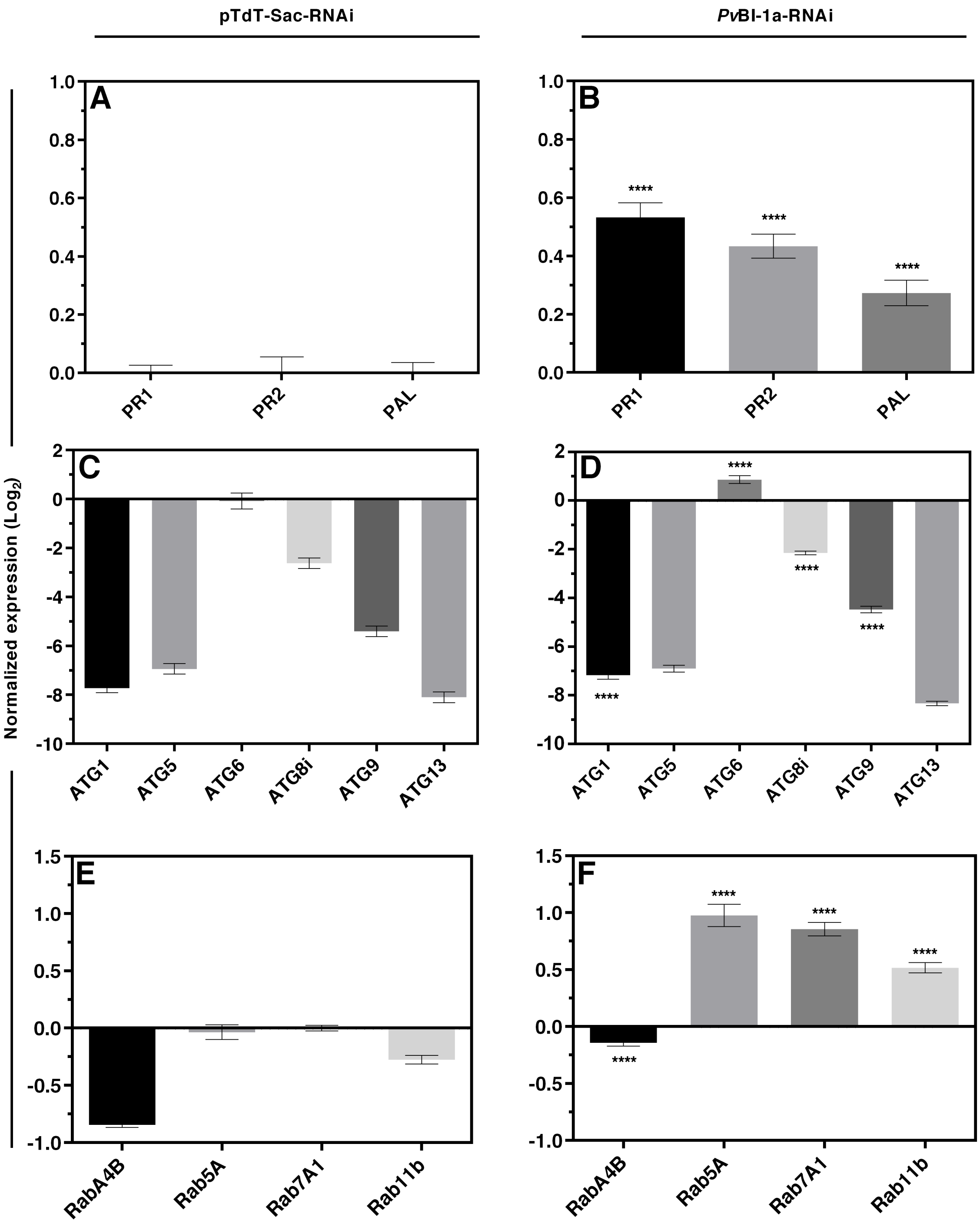

pTdT-Sac-RNAi
PvBI-1a-RNAi
Normalized expression (Log2)
A
B
C
D
E
F
PR1
PR2
PAL
ATG1
ATG5
ATG6
ATG8i
ATG9
ATG13
RabA4B
Rab5A
Rab7A1
Rab11b
****

Fig. S7
